# Supplementary material for: Intestinal Barrier Dysfunction and Microbial Translocation in Patients with First-Diagnosed Atrial Fibrillation
Source: Biomedicines. 2023 Jan 10;11(1):176. doi: 10.3390/biomedicines11010176 (PMC9856173; doi:10.3390/biomedicines11010176)
Supplement: Supplementary file 1 [file biomedicines-11-00176-s001.zip › biomedicines-2127316-supplementary.pdf]

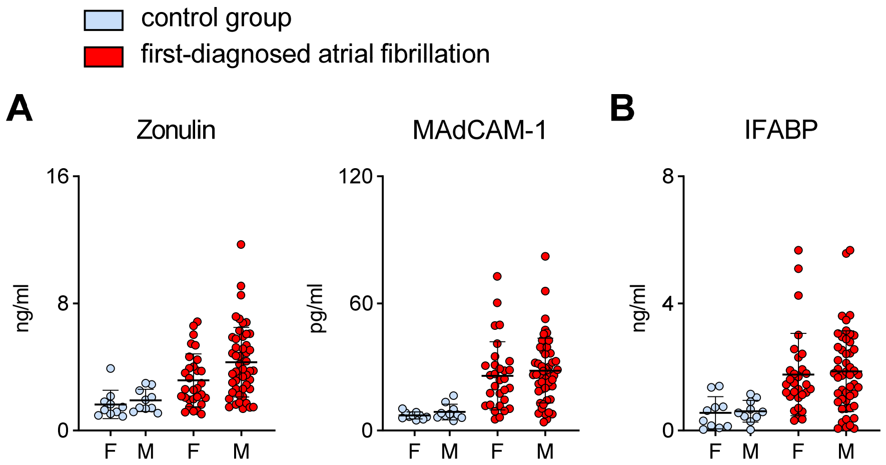

Figure S1. Consideration of gender-specific aspects. F, female; M, male. Intestinal barrier dysfunction in patients with first-diagnosed AF. Elevated circulating biomarkers suggestive of intestinal inflammation (**A**) (zonulin and mucosal endothelial cell adhesion molecule, MAdCAM-1) and enterocyte damage (**B**) (intestinal fatty acid binding protein, IFABP) that are associated with abnormal intestinal permeability. Patients with first-diagnosed AF (n = 80) were compared to controls (patients with chronic cardiovascular diseases but without AF) (n = 20). Results are expressed as single values, mean ± SD.

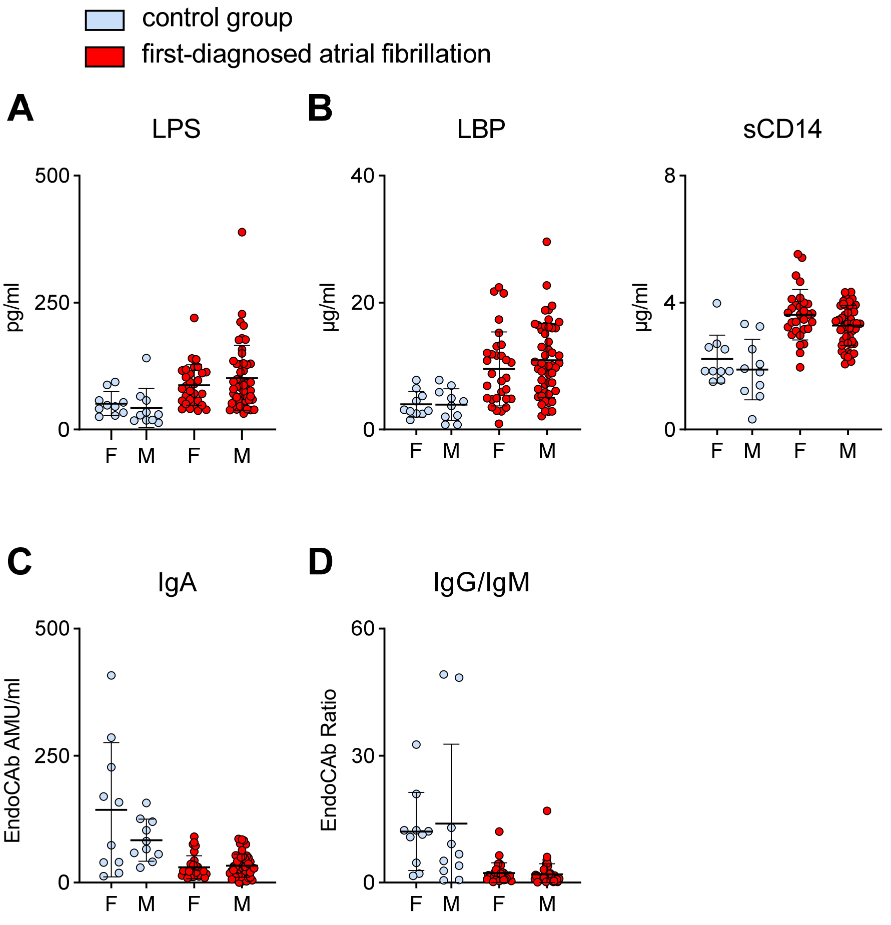

Figure S2. Consideration of gender-specific aspects. F, female; M, male. Biomarkers suggest the elevation of circulating gut-derived endotoxin in patients with first-diagnosed AF. Concentrations of markers for increased microbial translocation in plasma were measured by ELISA. Elevated levels of direct (lipopolysaccharide, LPS) (**A**) and indirect (LPS-binding protein, LBP; soluble CD14, sCD14) (**B**) indicators of low-grade endotoxaemia were present in plasma during early AF. Lower titres of circulating endotoxin core IgA antibodies (EndoCAb) (**C**) and a reduced ratio of EndoCAb IgG/IgM titres (**D**) in the AF cohort compared to controls. Results are expressed as single values (n = 20/80), mean ± SD.

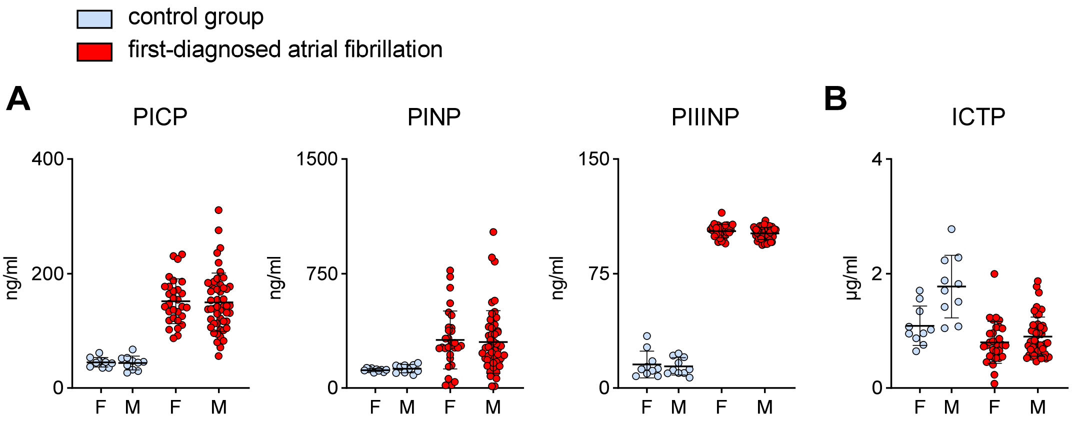

Figure S3. Consideration of gender-specific aspects. F, female; M, male. Increased plasma markers of collagen turnover in patients with first-diagnosed AF. Elevated surrogate markers of collagen synthesis (procollagen I C-terminal propeptide, PICP; procollagen I N-terminal propeptide, PINP; procollagen III N-terminal propeptide, PIIINP) (**A**) and reduced collagen degradation fragments (C-telopeptide of type I collagen, ICTP) (**B**) in the plasma of early AF patients (n = 80) vs. controls (n = 20). Results are expressed as single values, mean ± SD.
